# Supplementary material for: Assessment of Factors Influencing Personal Exposure to Air Pollution on Main Roads in Bogota: A Mixed-Method Study
Source: Medicina (Kaunas). 2022 Aug 19;58(8):1125. doi: 10.3390/medicina58081125 (PMC9416028; doi:10.3390/medicina58081125)
Supplement: Supplementary file 1 [file medicina-58-01125-s001.zip › medicina-1830946-supplementary.pdf]

Supplementary Matterials

Supplementary Table S1. Main characteristics of the monitored routes

Route/Location	Quality air condition IBOCA*	Mean Distance traveled	Bicycle infrastructure Cycle route	Characteristics of the route
**Southern Highway**	Low	6.8 kilometers	On the sidewalk	Main route with very high traffic flow
**Cali Avenue**	Regular and low	7.5 kilometers	On the sidewalk	Main route with very high traffic flow
**Quinto Centenario AVE**	Regular	8.7 kilometers	Next to the vehicular lane	Main route with moderate traffic flow
**116th Street**	Medium and good	6.1 kilometers	On the sidewalk and in the middle of the road.	Main route with high traffic flow

*Bogota Air Quality Index for its initials in Spanish (IBOCA).

Supplementary Table S2. Mean concentrations of $PM_{2.5}$ in by route and source compared with mean $PM_{2.5}$ concentrations from RCMB

Source	116th Street	Cali Avenue	Quinto Centenario	Southern Highway
ITHACA	15.66 ugm^{-3}	54.65 ugm^{-3}	36.24 ugm^{-3}	54.64 ugm^{-3}
RCMB	13.25 ugm^{-3}	23.23 ugm^{-3}	14.81 ugm^{-3}	25.65 ugm^{-3}

Proportion	1.18	2.35	2.45	2.13

Supplementary Table S3. MET’s* by transport mode

Mode	Mean	Median	SD	p
Minivan	1.463	1.455	0.025	0.9
Bike	1.668	1.598	0.270	0.008
Bus	1.506	1.487	0.058	0.17

***MET= metabolic equivalent of task**

Supplementary Table S4. Inhaled dose by the mode of transportation used by the participants.

Mode	Mean (μg)	Median (μg)	SD (μg)	Proportion of the daily dose inhaled during the trip
Minivan	6.76	5.93	5.21	0.02
Bike	16.41	10.58	18.7	0.08
Bus	12.17	7.98	13.18	0.05

Supplementary Table S5. Multivariate model Post $FEF_{25-75\%}$

Route	RR*	CI 95%		p
Southern Highway	1.011	0.493	2.072	0.977
Cali Avenue	1.692	0.827	3.461	0.150
V Centenario Avenue	1.486	0.165	5.306	0.19

116 Street	1			
**Mode**				
Minivan	1.214	0.658	2.240	0.534
Bike	0.862	0.459	1.618	0.644
Bus	1			
Inhaled $PM_{2.5}$ dose	0.978	0.956	1.000	0.360

**Poisson* regression model showing that the participants who made the journey by V Centenario avenue had 2.4 times the risk of having a reduction in the $FEF_{25-75\%}$ compared with 116th street.

Supplementary Table S6. Emergent categories and definitions.

Category	Subcategory	Definition
**Knowledge of air quality and its relationship with health**	**Pollution levels**	Qualitative individual assessment that defines the characteristics of air quality, the elements that are considered as generators of pollution, the substances that are identified in the environment and that are related to unwanted effects or that have a negative effect.
	**Health effects**	Changes in health resulting from exposure to sources of pollution or in polluted environments.
	**Effects on quality of life**	Changes in conditions that contribute to personal and social well-being.
**Attitudes towards exposure**	**Attitudes towards the Exposition**	Orientation and/or posture (positive or negative) when exposed to air pollutants or in polluted environments.
**Practices towards exposure**	**Protection against exposure**	Activities or behaviors of care and prevention in the face of exposure to contaminants or in contaminated environments.

**Reduction actions**	Activities to minimize pollution and improve air quality.
**Actions as a collective subject**	Behaviors that express the recognition of the environmental conditions of the environment and the historical, political, social, economic, and cultural dimensions and conditions, and from there the proposal of a change or a transformation based on the protection and/or defense of individual and collective interests

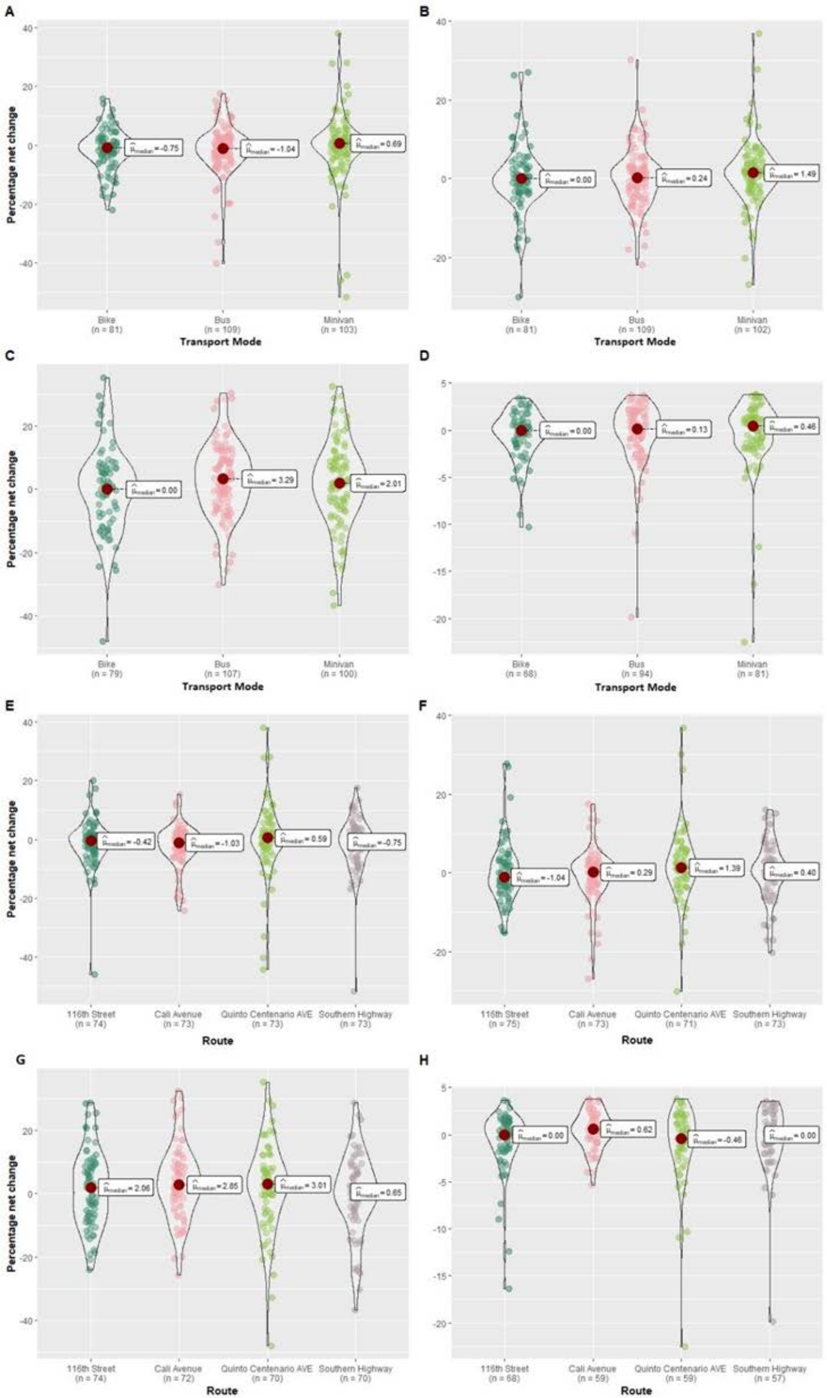
A
B
C
D
E
F
G
H
Percentage net change
Transport Mode
Route
Bike (n = 81)
Bus (n = 109)
Minivan (n = 103)
Minivan (n = 102)
Bike (n = 79)
Bus (n = 107)
Minivan (n = 100)
Bike (n = 68)
Bus (n = 94)
Minivan (n = 81)
116th Street (n = 74)
Cali Avenue (n = 73)
Quinto Centenario AVE (n = 73)
Southern Highway (n = 73)
116th Street (n = 75)
Quinto Centenario AVE (n = 71)
Cali Avenue (n = 72)
Quinto Centenario AVE (n = 70)
Southern Highway (n = 70)
116th Street (n = 68)
Cali Avenue (n = 59)
Quinto Centenario AVE (n = 59)
Southern Highway (n = 57)
$\hat{\mu}_{median}$ = -0.75
$\hat{\mu}_{median}$ = -1.04
$\hat{\mu}_{median}$ = 0.69
$\hat{\mu}_{median}$ = 0.00
$\hat{\mu}_{median}$ = 0.24
$\hat{\mu}_{median}$ = 1.49
$\hat{\mu}_{median}$ = 0.00
$\hat{\mu}_{median}$ = 3.29
$\hat{\mu}_{median}$ = 2.01
$\hat{\mu}_{median}$ = 0.00
$\hat{\mu}_{median}$ = 0.13
$\hat{\mu}_{median}$ = 0.46
$\hat{\mu}_{median}$ = -0.42
$\hat{\mu}_{median}$ = -1.03
$\hat{\mu}_{median}$ = 0.59
$\hat{\mu}_{median}$ = -0.75
$\hat{\mu}_{median}$ = -1.04
$\hat{\mu}_{median}$ = 0.29
$\hat{\mu}_{median}$ = 1.39
$\hat{\mu}_{median}$ = 0.40
$\hat{\mu}_{median}$ = 2.06
$\hat{\mu}_{median}$ = 2.65
$\hat{\mu}_{median}$ = 3.01
$\hat{\mu}_{median}$ = 0.65
$\hat{\mu}_{median}$ = 0.00
$\hat{\mu}_{median}$ = 0.62
$\hat{\mu}_{median}$ = -0.46
$\hat{\mu}_{median}$ = 0.00

Supplementary Figure S1. Changes in spirometry patterns by transport mode and route

It is estimated that the value of the PRE spirometry is 100%, it is calculated how much the POS net value of the spirometry represents in percentage compared to the PRE, the percentage delta between the POS and the PRE is obtained. The figure shows on the X axis the mode or route and on the Y axis the percentage delta stratified by: A y E = Forced vital capacity, B Y F = Forced expiratory volume in 1 second, C y G= Forced expiratory flow at 25 and 75% of the pulmonary volume, D y H = FEV1/FCV ratio

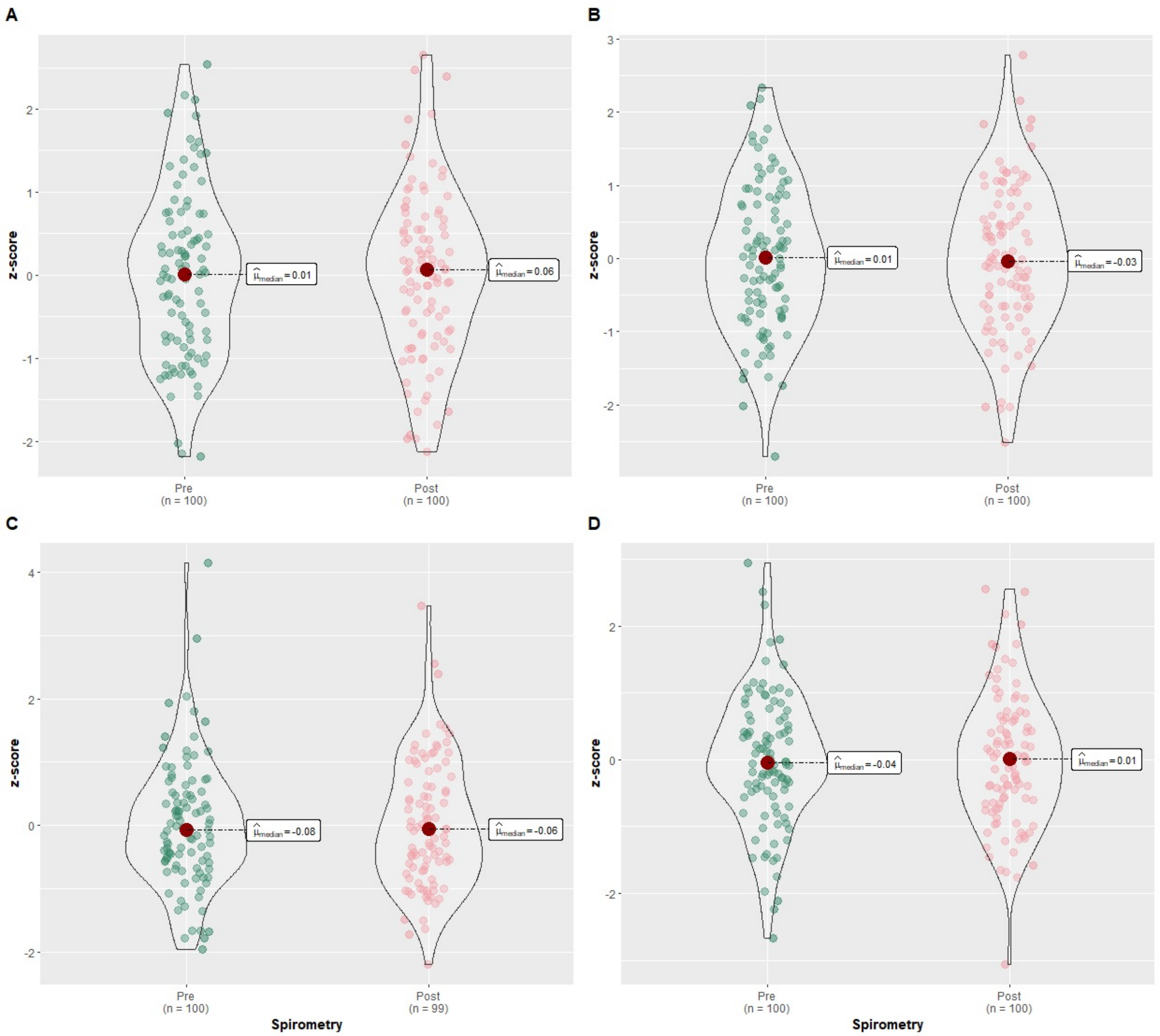

A
B
C
D
z-score
$\hat{\mu}_{median}$ = 0.01
$\hat{\mu}_{median}$ = 0.06
$\hat{\mu}_{median}$ = 0.01
$\hat{\mu}_{median}$ = -0.03
$\hat{\mu}_{median}$ = -0.08
$\hat{\mu}_{median}$ = -0.06
$\hat{\mu}_{median}$ = -0.04
$\hat{\mu}_{median}$ = 0.01
Pre (n = 100)
Post (n = 100)
Post (n = 99)
Spirometry

Supplementary Figure S2. Changes in the spirometry parameters (male): We have considered the spirometry variables (pre and post) for male and we estimate the z-score ($z = (x - \mu)/\sigma$) for each variable (a. Forced vital capacity, b.Forced expiratory volume in the first second, c. Forced expiratory flow at 25 and 75% of the pulmonary volume, d. FEV_1/FCV ratio).

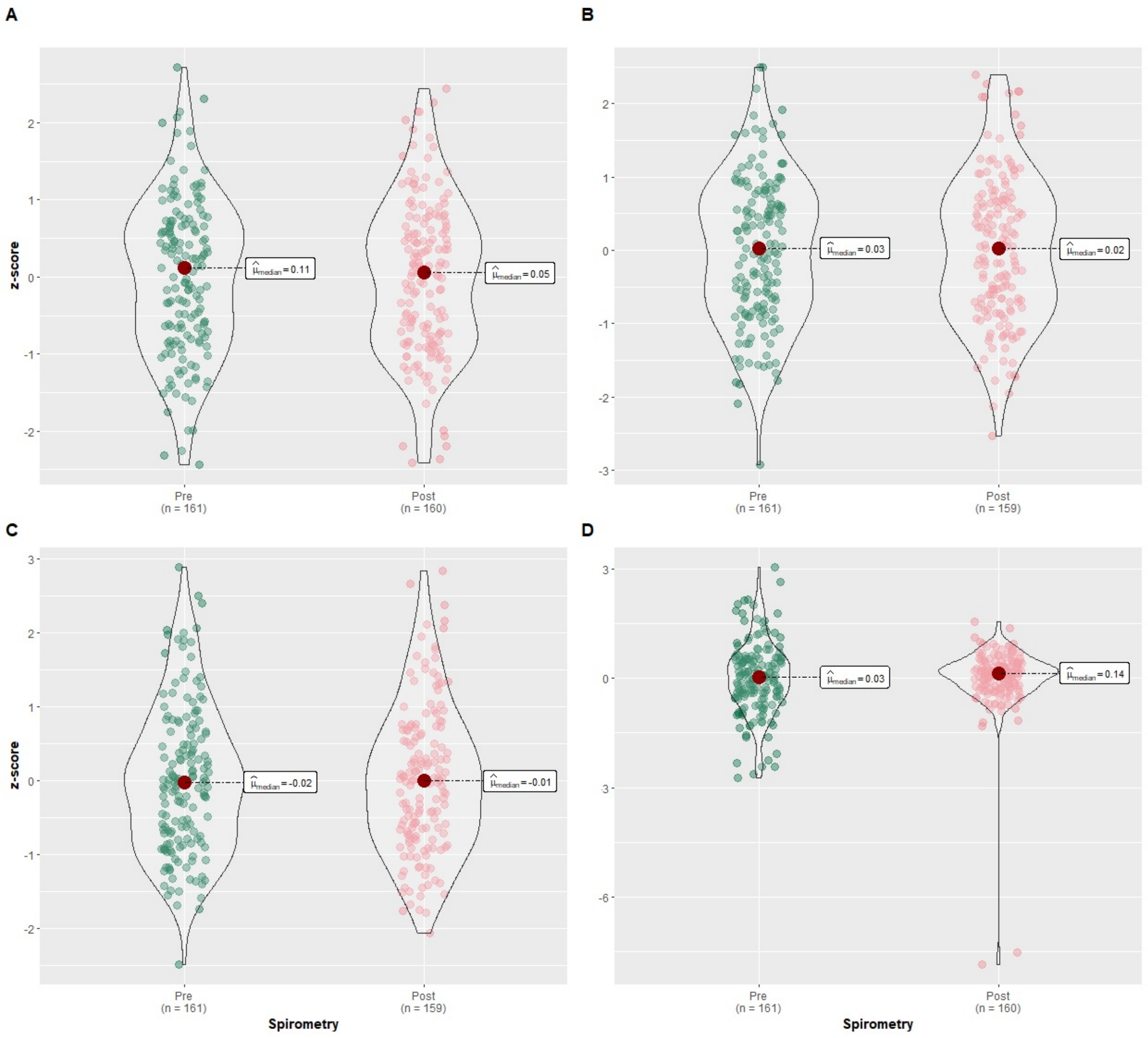

A
B
C
D
z-score
$\hat{\mu}_{median} = 0.11$
$\hat{\mu}_{median} = 0.05$
$\hat{\mu}_{median} = 0.03$
$\hat{\mu}_{median} = 0.02$
$\hat{\mu}_{median} = -0.02$
$\hat{\mu}_{median} = -0.01$
$\hat{\mu}_{median} = 0.03$
$\hat{\mu}_{median} = 0.14$
Pre (n = 161)
Post (n = 160)
Pre (n = 161)
Post (n = 159)
Pre (n = 161)
Post (n = 159)
Pre (n = 161)
Post (n = 160)
Spirometry

Supplementary Figure S3. Changes in the spirometry parameters (female). We have considered the spirometry variables (pre and post) for females and we estimate the z-score ($z = (x - \mu)/\sigma$) for each variable (a. Forced vital capacity, b.Forced expiratory volume in the first second, c. Forced expiratory flow at 25 and 75% of the pulmonary volume, d. FEV_1/FCV ratio.)

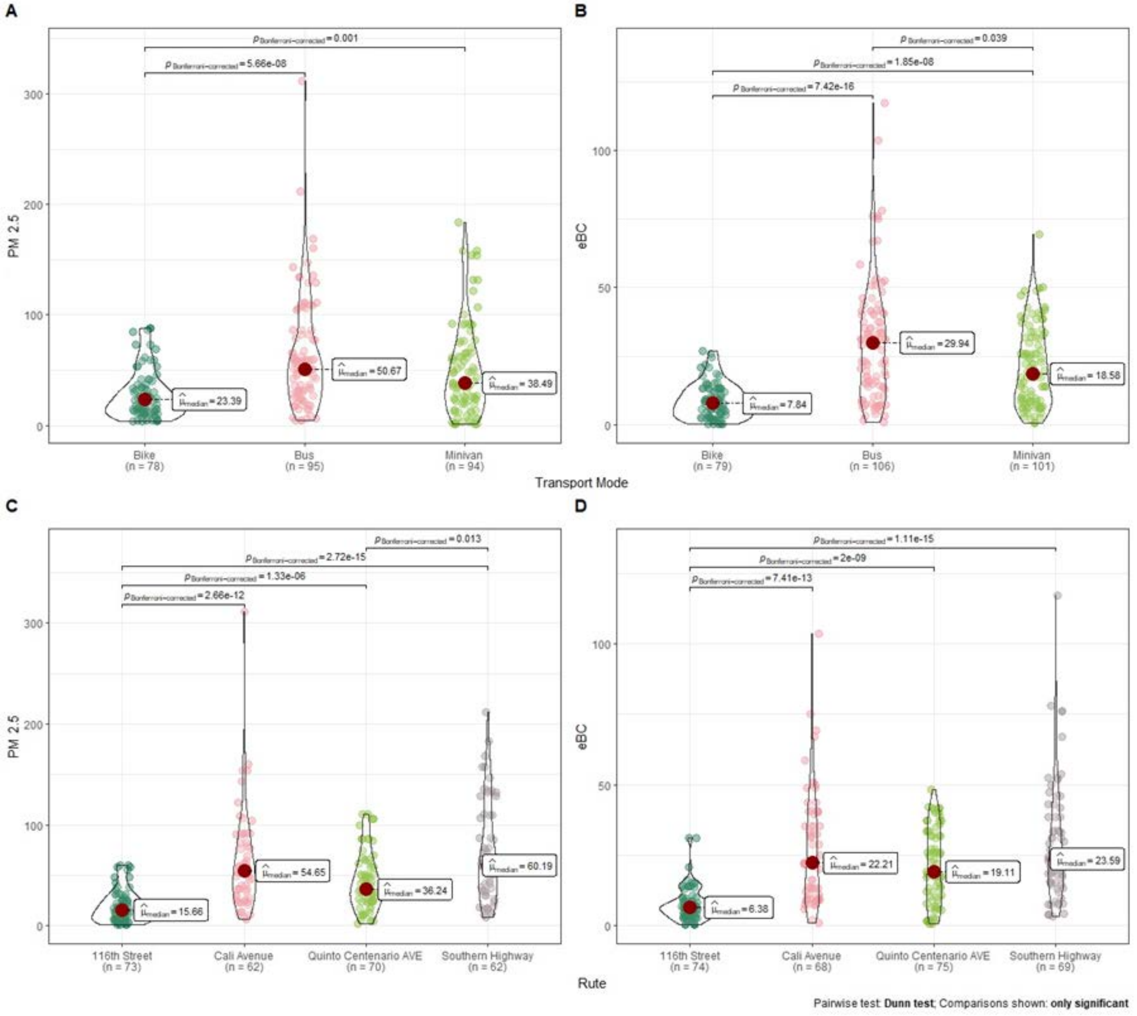
A
B
C
D
PM 2.5
eBC
$p_{Bonferroni-corrected}$ = 0.001
$p_{Bonferroni-corrected}$ = 5.66e-08
$\hat{\mu}_{median}$ = 23.39
$\hat{\mu}_{median}$ = 50.67
$\hat{\mu}_{median}$ = 38.49
Bike (n = 78)
Bus (n = 95)
Minivan (n = 94)
$p_{Bonferroni-corrected}$ = 0.039
$p_{Bonferroni-corrected}$ = 1.85e-08
$p_{Bonferroni-corrected}$ = 7.42e-16
$\hat{\mu}_{median}$ = 7.84
$\hat{\mu}_{median}$ = 29.94
$\hat{\mu}_{median}$ = 18.58
Bike (n = 79)
Bus (n = 106)
Minivan (n = 101)
Transport Mode
$p_{Bonferroni-corrected}$ = 0.013
$p_{Bonferroni-corrected}$ = 2.72e-15
$p_{Bonferroni-corrected}$ = 1.33e-06
$p_{Bonferroni-corrected}$ = 2.66e-12
$\hat{\mu}_{median}$ = 15.66
$\hat{\mu}_{median}$ = 54.65
$\hat{\mu}_{median}$ = 36.24
$\hat{\mu}_{median}$ = 60.19
116th Street (n = 73)
Cali Avenue (n = 62)
Quinto Centenario AVE (n = 70)
Southern Highway (n = 62)
$p_{Bonferroni-corrected}$ = 1.11e-15
$p_{Bonferroni-corrected}$ = 2e-09
$p_{Bonferroni-corrected}$ = 7.41e-13
$\hat{\mu}_{median}$ = 6.38
$\hat{\mu}_{median}$ = 22.21
$\hat{\mu}_{median}$ = 19.11
$\hat{\mu}_{median}$ = 23.59
116th Street (n = 74)
Cali Avenue (n = 68)
Quinto Centenario AVE (n = 75)
Southern Highway (n = 69)
Rute
Pairwise test: Dunn test; Comparisons shown: only significant

Supplementary Figure S4. Concentrations of $PM_{2.5}$ and eBC pollutants in the microenvironments. The red dots indicate the median value of the concentration of pollutants by transport mode and route. The Y axis shows the values of pollutant concentrations in ugm^{-3}. A) $PM_{2.5}$ concentration ug m^{-3} by transport mode. B) Black carbon concentration ugm^{-3} by transport mode. C) $PM_{2.5}$ Concentration ug m^{-3} by route. D) Black carbon concentration ugm^{-3} by route.

A.

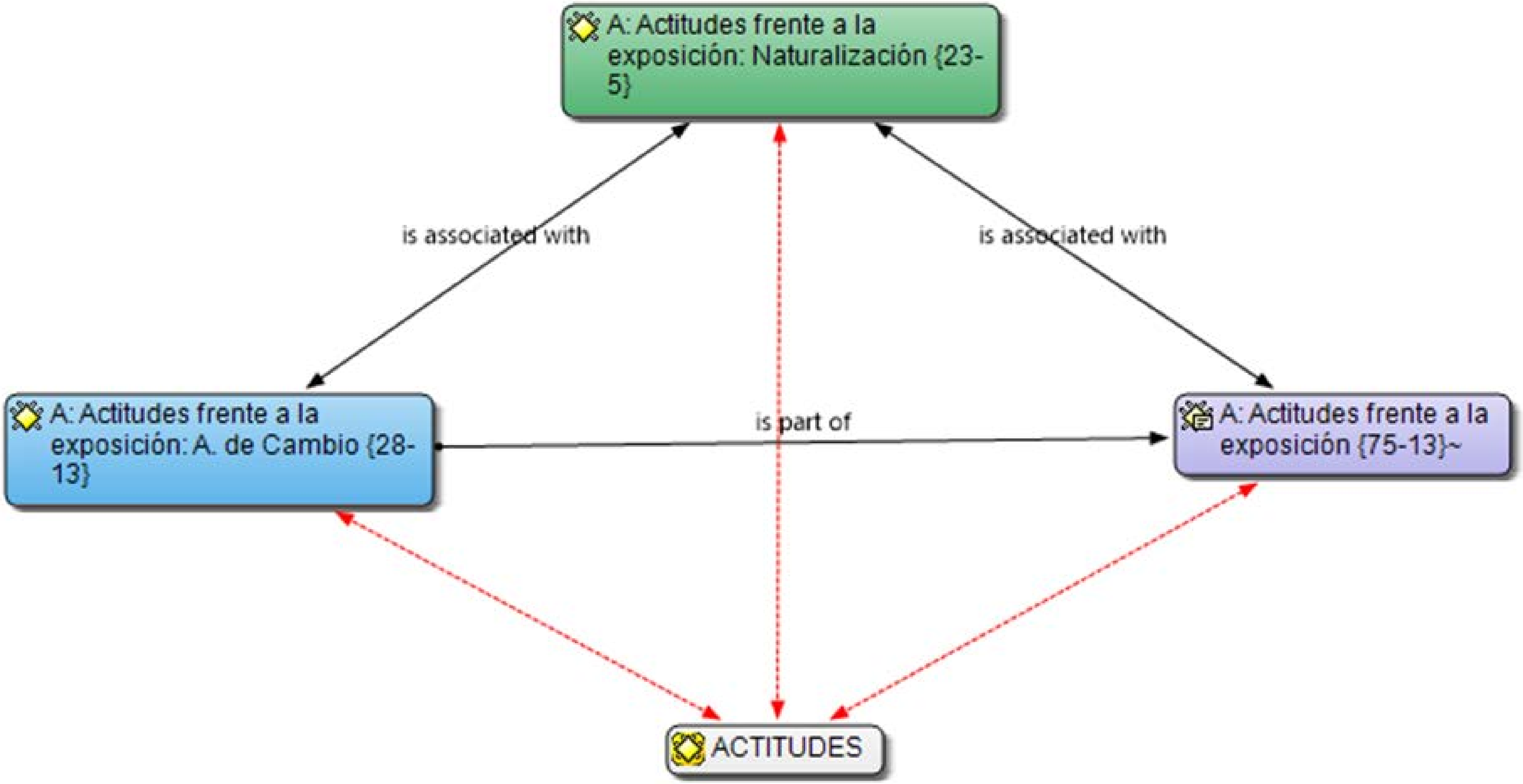
A: Actitudes frente a la exposición: Naturalización {23-5}
is associated with
is associated with
A: Actitudes frente a la exposición: A. de Cambio {28-13}
is part of
A: Actitudes frente a la exposición {75-13}~
ACTITUDES

B.

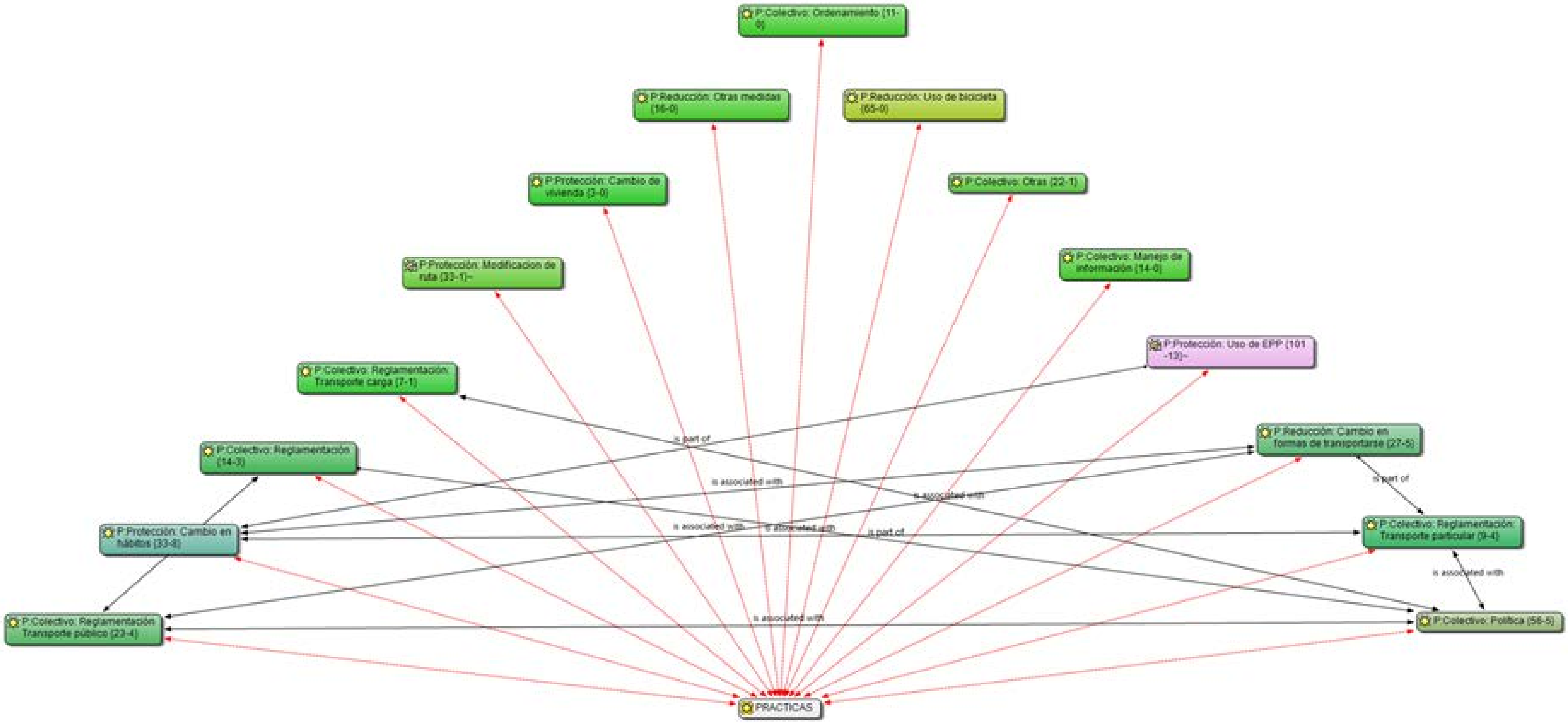

P:Colectivo: Ordenamiento (11-0)
P:Reducción: Otras medidas (16-0)
P:Reducción: Uso de bicicleta (65-0)
P:Protección: Cambio de vivienda (3-0)
P:Colectivo: Otras (22-1)
P:Protección: Modificacion de ruta (33-1)~
P:Colectivo: Manejo de información (14-0)
P:Protección: Uso de EPP (101-13)~
P:Colectivo: Reglamentación: Transporte carga (7-1)
P:Reducción: Cambio en formas de transportarse (27-5)
P:Colectivo: Reglamentación (14-3)
P:Protección: Cambio en hábitos (33-8)
P:Colectivo: Reglamentación: Transporte particular (9-4)
P:Colectivo: Reglamentación: Transporte público (23-4)
P:Colectivo: Política (56-5)
PRACTICAS
is part of
is associated with

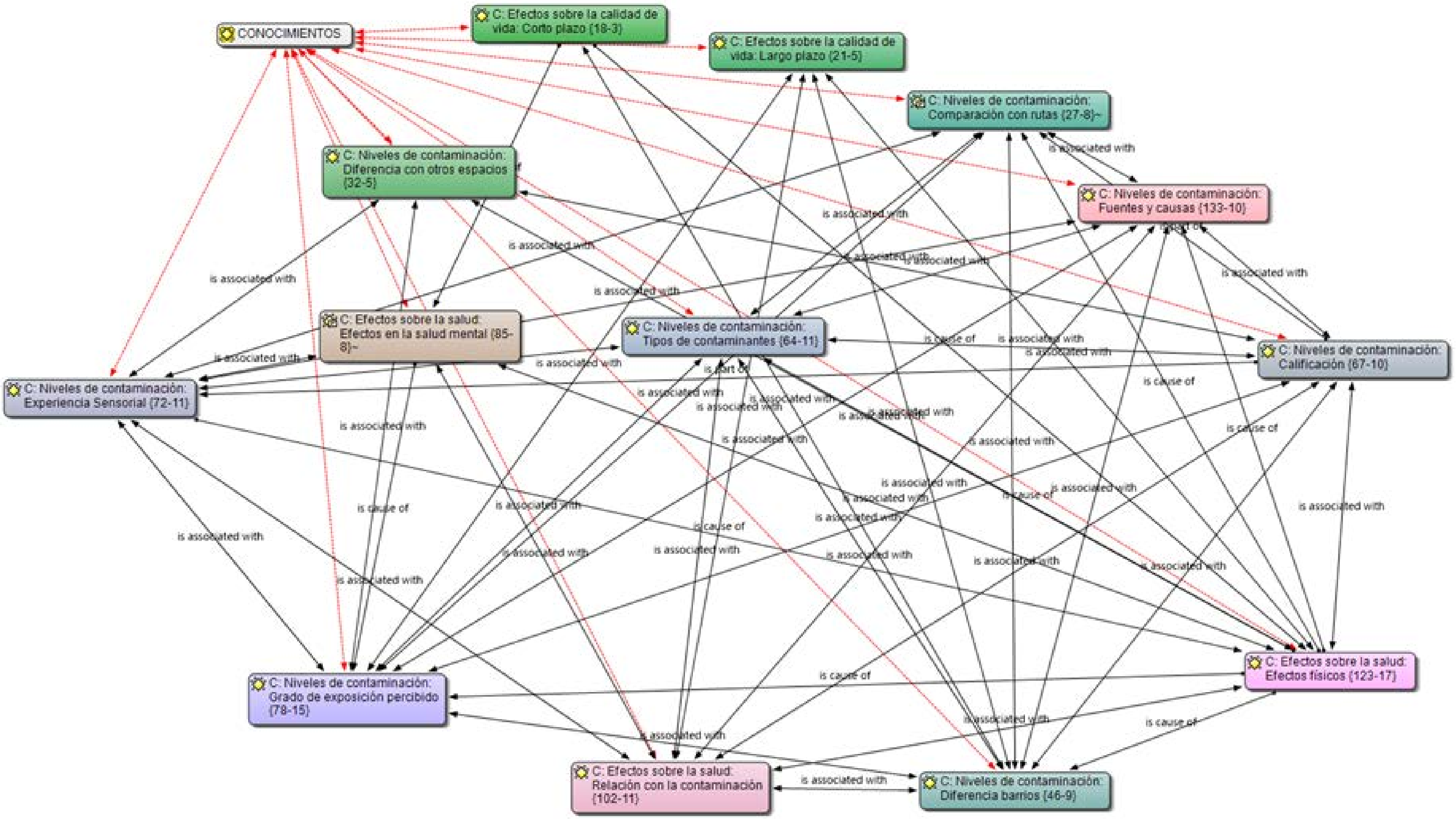

C.

Supplementary Figure S5. Code trees. A. Qualitative analysis of Knowledge, B. Qualitative analysis of Attitudes. C. Qualitative analysis of Practices.

The code tree is built from the coding process where the text is divided into units of meaning in social contexts that express a collective representation. These units materialize in codes that constitute the axis of analysis and from which association or contradiction relationships are established that can be identified in the graph through the links or arrows between codes. Thus, the red arrows indicate the codes associated with the category, and the black arrows the association between codes. On the other hand, the network that is generated is determined by the rationale, that is, the number of citations associated with each code, and by the density, which refers to the number of relationships that one code has with others. Thus, in the graphs, the color of the nodes is determined by the foundation and by the density that can be identified in the numbers that are in parentheses. For example, the code Health Effects: Physical Effects (123-17) has 123 associated citations (rationale) and is related to 17 codes (density). Finally, the networks are in the original language of the research so as not to alter their meaning of interpretation
